# Meso Hybridized Silk Fibroin Watchband for Wearable Biopotential Sensing and AI Gesture Signaling

**DOI:** 10.1002/advs.202410702

**Published:** 2024-12-11

**Authors:** Xiao Wang, Changsheng Lu, Zerong Jiang, Guangwei Shao, Jingzhe Cao, Xiang Yang Liu

**Affiliations:** ^1^ State Key Laboratory of Marine Environmental Science (MEL) College of Ocean and Earth Sciences Xiamen University Xiamen Fujian 361102 P. R. China; ^2^ Engineering Research Center of Technical Textiles Ministry of Education College of Textiles Donghua University Shanghai 201620 P. R. China; ^3^ College of Textile and Garment Shaoxing University Shaoxing Zhejiang 312000 P. R. China

**Keywords:** flexible AI watchband, meso dry electrode, Mo‐Au filament network, silk fibroin

## Abstract

Human biopotential signals, such as electrocardiography, are closely linked to health and chronic conditions. Electromyography, corresponds to muscle actions and is pertinent to human‐machine interactions. Here, we present a type of smart and flexible watchband that includes a mini flexible electrode array based on Mo‐Au filament mesh, combined with mesoscopic hybridized silk fibroin films. As the layer in contact with the skin, waterborne polyurethane and SF create a highly flexible and permeable meso‐hybridized SF/WPU layer, ensuring skin‐friendliness and comfortable wearing. The flexible FM electrodes are created by integrating Mo‐Au FM into 2D‐interconnected networks. Molybdenum filaments provide high rigidity and are coated with Aurum to enhance conductivity. The use of Mo‐Au FMs in warp‐knitted patterns results in high SNR (43.22 dB), high sensitivity (44.43 mV/kg), and significant motion noise reduction due to the pattern's elastic deformability and skin‐gripping properties. Leveraging these unique technologies, these smart watchbands excel in prolonged sensing operation, grasping force detection, and gesture recognition. Through smart raining via deep learning, we achieved an unparalleled recognition rate (96% across 20 volunteers of different genders) among other EMG sensing devices. These results have significant implications for human‐machine interaction, including applications in underwater robot control, drone operation, and autonomous vehicle control.

## Graphical abstract

X. Wang et al. present a smart watchband integrating advanced materials and deep learning, enhancing wearable health tech. This AI‐based device demonstrates exceptional sensitivity, comfort, durability, and accurate hand gesture recognition, surpassing previous standards with innovative technologies.

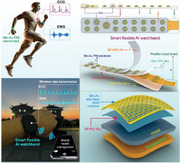

## 1 Introduction

Human biopotential signals such as electrocardiography (ECG) and electromyography (EMG) are pivotal in diagnosing and managing conditions related to the heart and muscles.^[^ ^1^ ^]^ These biopotentials are traditionally captured through epidermal electrodes that electrically interface with the skin. Accurate and continuous monitoring of these signals is essential, particularly for detecting subtle and chronic conditions in everyday settings outside clinical environments.^[^ ^2^ ^]^ Human‐machine interaction (HMI), which refers to how humans interact with computer systems, has gradually become an integral part of daily life.^[^ ^3^ ^]^ Among the various forms of HMI, EMG‐based control plays a key role by allowing users to control computers and external devices through muscle electrical signals.^[^ ^4^ ^]^ Currently, EMG control technology has been widely applied in various fields such as virtual reality (VR),^[^ ^5^ ^]^ gaming,^[^ ^6^ ^]^ assistive devices,^[^ ^7^ ^]^ and healthcare.^[^ ^8^ ^]^ However, this technology still faces several challenges.^[^ ^9^ ^]^ The acquisition and processing of EMG signals require high‐precision sensors and complex signal‐processing algorithms, which increase system costs and complexity.^[^ ^10^ ^]^ EMG signals are susceptible to interference and noise due to factors such as muscle activity, electrode placement, and external disturbances, affecting the stability and accuracy of the system.^[^ ^11^ ^]^ Therefore, improving the efficiency of EMG signal acquisition and processing, reducing system costs, and enhancing system stability and accuracy are major challenges facing current EMG control technology. Among these challenges, the performance of electrodes is particularly critical. Ag/AgCl gel electrodes are commonly used but have notable drawbacks, including signal degradation over time due to gel electrolyte evaporation, discomfort, and skin irritation during prolonged use.^[^ ^12^ ^]^ In response, there has been a shift toward developing skin‐friendly, dry electrodes, categorized mainly into impedance and capacitive types, each utilizing different materials such as metals, conductive polymer composites, and intrinsically conductive polymers.^[^ ^13^ ^]^ Nevertheless, the current dry electrode techniques suffer from a low sensitivity and signal‐to‐noise ratio (SNR). Therefore, a new type of flexible dry electrodes needs to be developed.

In this work, we integrate Mo‐Au FM electrodes into a skin‐friendly cocoon silk hybrid material‐based watchband and employ a deep‐learning algorithm using convolutional neural networks (CNN) to acquire an AI‐sensing watchband. Metals are frequently chosen as electrode materials due to their excellent electric conductivity. However, the major drawbacks include rigidity, lack of wearing comfort, and susceptibility to motion noise during movement. In contrast, metal filament textile electrodes are attractive due to their excellent conductivity and corrosion resistance.

To overcome these limitations, we focus on flexible Mo‐Au filament meshes, where Mo‐filaments are coated with Au (Mo‐Au FM electrodes). This design enhances the angular deformation restoration elasticity, conductivity, and skin‐gripping properties of mesh networks. With buffering provided by polydimethylsiloxane (PDMS) filling, the Mo‐Au FM flexible electrodes demonstrate high performance and comfortable wearability. Combined with meso‐hybridized silk fibroin (SF) and waterborne polyurethane (WPU), we aim to develop high‐performance, comfortable flexible watchbands. These smart watchbands seamlessly adapt to the wearer's movements and can accurately recognize a variety of gestures with high precision.

## 2 Results and Discussion

### 2.1 Design of Flexible AI Watchbands

**Figure 1:**
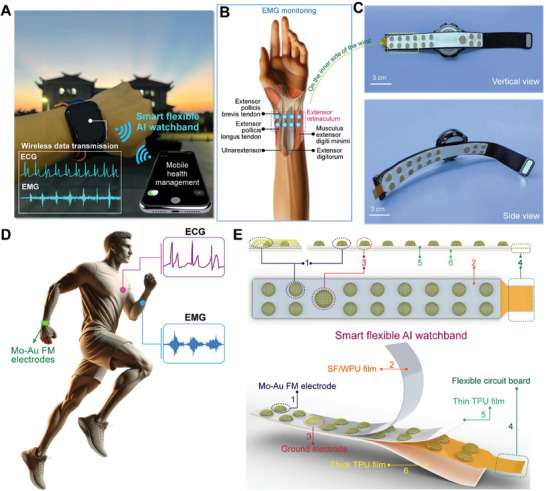
Illustration of flexible smart watchband, including the detailed structure and the potential applications. A) Illustration of a smartwatch with a flexible smart band in real‐time biopotential monitoring. The system is capable of multi‐channel recording of ECG and EMG signals. B) An anatomical illustration of a hand, highlighting the muscles and tendons relevant to EMG monitoring. Blue dots indicate the placement of Mo‐Au FM electrodes on the inner side of the wrist, covering key anatomical structures such as the extensor pollicis longus tendon, extensor pollicis brevis, ulnar extensor of the wrist, extensor retinaculum, extensor of the little finger, and the muscle lateral to the extensor digiti minimi. Importantly, the ground electrode (3) is placed on the styloid process of the ulna at the wrist to reduce noise and interference, thereby enhancing the quality of the EMG signals. The ulna styloid process, a prominent bone landmark, is relatively close to the muscle groups being tested. Additionally, its location aids in stabilizing the electrode, ensuring optimal contact. C) Photos of the smart flexible watchband. Scale bars, 3 cm. D) Three different views of the smart flexible watch band: 1: 2×8 channels of flexible dry Mo‐Au FM electrodes. 2: Water vapor permeability and skin‐friendly meso hybridized SF/WPU film. 3: Ground layer. 4: Flexible circuit board. 5:Thin TPU film. 6: Thick TPU film.

The flexible AI watchbands are designed to sense ECG/EMG signals and recognize hand gestures in HMI, as illustrated in **Figure** **1**. In addition to offering high accuracy, sensitivity, and SNR, the watchbands are designed for comfortable wear, utilizing an array of flexible dry electrodes embedded in skin‐friendly materials. Figure 1A shows an overview of these watchbands. A flexible wearable watchband includes a single flexible patch with 2×8 Mo‐Au FM working electrodes and a ground electrode for measuring ECG/EMG signals (Figure 1B‐C). The blue dots in Figure 1B indicate the placement of Mo‐Au FM electrodes on the inner wrist, covering key anatomical structures,^[^ ^14^ ^]^ such as the extensor pollicis longus tendon, extensor pollicis brevis, ulnar extensor of the wrist, extensor retinaculum, extensor of the little finger, and the muscle lateral to the extensor digit minimi. Importantly, the ground electrode is strategically placed on the styloid process of the ulna to reduce noise and interference, thereby enhancing the quality of the EMG signals. The ulna styloid process, a prominent bone landmark, is located near the muscle groups being tested,^[^ ^15^ ^]^ and its positioning helps stabilize the electrode, ensuring optimal contact.

ECG signals are key indicators of heart health and circulatory diseases. The soft and unobtrusive watchbands offer a more convenient alternative to traditional wearable biopotential monitors, seamlessly integrating with the skin to ensure accurate and consistent signal detection, even during movement. Figure 1C displays photos of the wearable smart flexible watchbands, including both vertical view and side view. Figure 1D illustrates the practical application of the watchband on a human subject, emphasizing the ease of monitoring biopotentials during exercise. This highlights the importance of the electrodes maintaining contact with the skin for accurate recording of biopotentials across a range of activities, from sedentary to high‐motion scenarios. The technical breakdown of the watchband in Figure 1E delves into the layered structure with an array of flexible Mo‐Au FM electrodes directly contacting the ECG and EMG acupoints on the wrist. The electrodes are embedded in the multi‐layer watchbands, where the top is the SF/PU meso‐hybridized layer, followed by thin thermoplastic PU (TPU) layers. This multi‐layered structure, along with the skin‐gripping properties of the Mo‐Au filaments, permits the electrode array to adapt to skin deformation and maintain signal quality, which is essential for reliable signal sensing and long‐term use.

### 2.2 Knitting Mo‐Au FM Flexible Electrodes and Pattern Shape Elasticity

**Figure 2:**
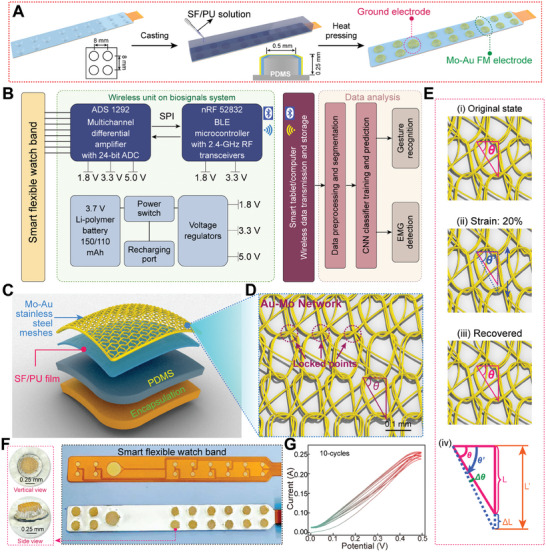
The architecture and fabrication of smart flexible watchband and electronic system. A) The design and fabrication of the smart flexible EMG watchband: First, the SF was mixed with WPU. The mixture was then cast onto a flat substrate and placed in an oven to dry, forming a uniform composite film. Subsequently, the prepared SF/WPU film was bonded to the Mo‐Au mesh through a hot‐pressing process. Next, the Mo‐Au mesh conductive layer was stamped to form a raised structure. Finally, a pre‐mixed PDMS solution was poured into the hollow raised structure of the metal mesh conductive layer and cured in an oven. B) The diagram illustration of the sequence from electrophysiological signals detection. C) The diagram of the structure of the 3D Mo‐Au FM electrode, includes Mo‐Au FM, flexible and skin‐friendly membrane (SF/WPU film), elastomer layer (PDMS), and encapsulation. D) The smart flexible watch band with the 3D Mo‐Au FM electrode. E) Images of Mo‐Au FM in various deformation states: (i) original; (ii) 20% deformation, and (iii) restored; (iv) stretched triangle model. F) Photos of the smart flexible watchband. The enlarged image of the 3D Mo‐Au FM electrode from the vertical view and the side view. G) Electric stability: the I‐V cycles of the Mo‐Au FM electrode after 10 cycles.

**Figure** **2** **A** presents the architecture and manufacturing procedures of the AI watchband. To create a multi‐layer structure, a combination of casting and thermoplastic manufacturing methods was employed, integrating elastic dry Mo‐Au FM electrodes with the smart watchband. This approach can handle various materials and achieve the creation of complex structures. The watchband comprises 2×8 arrays of dry Mo‐Au FM electrodes, spaced 10 mm apart, with each electrode having a height of 0.25 mm and a diameter of ≈0.5 mm, along with ground and reference electrodes. This design provides the structural foundation for enhancing gesture recognition accuracy and precision. The diagram in Figure 2B illustrates the overall flow of data measurement and analysis. The intricate construction of the dry Mo‐Au FM electrodes, shown in Figure 2C, demonstrates careful consideration of biocompatibility and durability. All electronic components are embedded in a soft fabric encapsulation, offering a nonstick dry surface and protecting them from mechanical strain.^[^ ^16^ ^]^ Additionally, the elastomer layer of polydimethylsiloxane (PDMS) enhances skin contact and ensures the skin‐gripping properties of the Mo‐Au filaments. Unlike one‐time‐use gel electrodes, the dry Mo‐Au FM electrodes offer reusability for multiple days of biopotential recording.^[^ ^17^ ^]^ By adhering to the requirements for dry electrodes in HMI, electrode positions for measuring ECG and EMG were carefully selected.

We notice that Mo filaments are rigid and tough, lacking extensibility. Using Mo filaments to fabricate electrodes would result in poor wearing comfort. Therefore, by leveraging the bending elasticity of Mo filaments, weaving them into a specific pattern may achieve adjustable pattern shape elasticity and skin‐gripping properties.

The mechanical properties of Mo‐Au filaments and twisted Mo‐Au filaments are analyzed as follows.^[^ ^18^ ^]^ Although metal filaments and plain textiles reported in the literature exhibit some stretchability, they tend to shear at an angle of 45°, which limits their overall stretchability.^[^ ^19^ ^]^ As shown in Figure 2C‐D, the Mo‐Au FM has a network‐like 2D structure of 1×1 loops. A locking point occurs, creating a triangular pattern that can be stretched and restored by altering the angle (*θ*) at a locking point.

The woven fabric structure is demonstrated in Figure S3B (Supporting Information), which consists of warp and weft threads. This plain‐woven fabric does not exhibit tensile recovery. Each filament has a size of ≈0.5 mm (Figure S3A, Supporting Information). In its natural state, a 3 cm×3 cm Mo‐Au FM template makes a hollow tube, demonstrating the softness and stretchability of the Mo‐Au FM. The Mo‐Au FM was knitted using a modified Raschel warp knitting machine, with ultrafine Mo‐Au FM having a diameter of 27 µm as the raw material.

**Figure 3:**
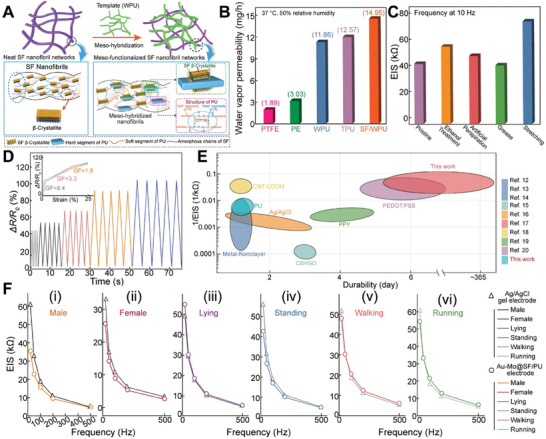
Mechanical, electrical, and durability performance of Mo‐Au FM electrode. A) Schematic of mesoscopic SF hybridization and interaction synergy of WPU with SF. The mesoscopic functional template (WPU) was added to the regenerated SF solution. B) The relationships between water vapor permeability and the placing time in an incubator at 37 °C, 50% relative humidity of polytetrafluoroethylene (PTFE) (rose bar), polyethylene (PE) (green bar), WPU (light blue bar), TPU (purple bar), and SF/WPU composite film (orange bar). C) EIS of Mo‐Au FM electrode under different treatments. D) Relative resistance changes of Mo‐Au FM as a function of strain. E) In comparison of durability and 1/EIS values with other dry electrodes, our Mo‐Au FM flexible electrodes are significantly longer (over 120 times longer) than other flexible dry or wet electrodes. F) (i‐ii) Static contact impedance of commercial gel electrode and Mo‐Au FM electrode between men and women. (iii‐vi) Contact impedance of commercial gel electrode and Mo‐Au FM electrode in different states.

The Mo‐Au FM network exhibits remarkable flexibility and elasticity (Figure S3E, Supporting Information) and can stretch up to 20% (Figure 3D; Figure S3D, Supporting Information). The 2D interconnected network structure of the Mo‐Au FM enabled the combining of metal fibers, with each half‐loop length contributing to low resistance. Due to these advantages, the Mo‐Au FM is a superior conductive substrate for high‐performance textile electrodes.

Figure 2E (i‐iii) present the changes in the Mo‐Au FM loops under small deformation (20%), showing the original, 20% deformation, and restored state. In each loop, the fiber bends into a triangle shape (Figure 2D: purple dashed circle). When the Mo‐Au FM is stretched vertically, the fiber slips and increases the angle between the slanted and the horizontal fiber in the triangle model.^[^ ^18b^ ^]^ According to our previous report, the triangle model is shown in Figure 2E‐iv, where *θ* is the original angle, *Δθ* is the increased angle, and *θ′* is the final angle.^[^ ^18b^ ^]^ *L*, *ΔL*, and *L′* are the corresponding side lengths of *θ*, *Δθ*, and *θ′*, respectively. The strain rate *ΔL/L* is defined by the equation:^[^ ^18b^ ^]^

(1) $$\mathrm{Strain}\;\mathrm{rate}=\Delta L/L=\tan\left(\Delta \theta +\theta \right)\;\tan\theta -1\;$$

The change in angle *Δθ* was about 6°, while the angle *θ* reached ≈44°. The calculated strain rate was ≈23%, which aligns with the actual observed strain rate of 20%. Once the applied force on the Mo‐Au FM was released, the angle shifted back from *θ′* to its original value *θ*, allowing the loops to largely regain their initial configuration. This structural design imparts the Mo‐Au FM with remarkable tensile recovery capabilities.

Figure 2F showcases the final assembly of the watchband, with a special emphasis on the low‐temperature soldering process that connects the electrodes to the conductive polyimide (PI) watchband. This step is crucial for maintaining the mechanical stability of the electrodes, ensuring the device remains functional and reliable over time. The durability of the Mo‐Au FM was evaluated through cyclic voltammetry tests, which electrical performance even after 10 cycles (Figure 2G). After ten cycles within the voltage range of 0‐0.5 V, the current of the Mo‐Au FM showed a minimal decrease, indicating its suitability as a long‐term functional layer for dry electrodes. Collectively, these figures delineate a comprehensive wearable system that harmoniously blends flexible electronics with advanced signal processing, paving the way for non‐intrusive, continuous biopotential monitoring.

To the rigidity and conductivity of Mo‐Au filaments and the particular weaving patterns of FM, the electrodes exert long‐lasting conductivity and the grasping ability to human skin. These are attributed to various superior performance of the electrodes in terms of sensitivity, SNR, low motional noise, etc.

### 2.3 Meso‐Hybridization of Silk Fibroin and PU Molecules for Skin‐Friendly Top Layer of Watchbands and Enhanced Overall Performance

As shown in Figure 1, the top layer in contact with human skin is derived from SF/WPU meso‐hybridization, where SF plays a transformative role. SF, a biopolymer known for its biocompatibility, mechanical strength, and flexibility, is especially in thin film form.^[^ ^20^ ^]^ These materials can be chemically and biologically functionalized, providing a range of physical properties.^[^ ^20^, ^21^ ^]^ To enhance water vapor permeability and skin‐friendliness, SF is hybridized with PU to form meso‐hybridized SF/PU films. Beyond biocompatibility, water vapor permeability is crucial for the long‐wear comfort of flexible watchbands, and the meso‐hybridization of SF and PU is designed to improve this performance.

To create a stable and elastic material from brittle regenerated SF films, we mesoscopically hybridized SF with WPU molecules (Figure S1, Supporting Information).^[^ ^20c^ ^]^ PU is a linear polymer with a backbone composed of alternately linked soft and hard segments.^[^ ^20^, ^22^ ^]^ The secondary structure of SF, primarily consisting of α‐helix and β‐sheet,^[^ ^23^ ^]^ significantly influences their performance.

During the hybridization process, intermolecular hydrogen bonding and van der Walls forces between PU and SF molecules—specifically the ‐C = O‐ and ‐NH‐ groups in PU and the carboxyl and amino groups in SF (Figure S2E, Supporting Information) —initiate the nucleation of β‐crystallites on the surface of the hard segments of PU molecules (Figure S2A‐D, Supporting Information).^[^ ^24^ ^]^ This interaction also attracts adjacent free SF molecules to PU seeds, increasing local concentration and triggering further β‐crystallite nucleation (Figure S2F, Supporting Information).^[^ ^25^ ^]^

Since the mesoscopic structure is the macroscopic of flexible materials, variations in the SF mesostructure through PU hybridization modify the mechanical properties.^[^ ^20^, ^22^ ^]^ As indicated by **Figure** **3** **A**, neat SF films have rigid β‐crystallites networks, making them relatively tough but brittle. Conversely, for SF‐PU hybridized materials, the non‐crystalline random coils within the soft segments of PU are incorporated into the β‐crystallites, enhancing flexibility and extensibility. Additionally, the entanglement between the soft segments of PU and SF molecular chains further improves the ductility of the hybrid materials.

Figure 3B illustrates the breathability of the smart watchbands, teated using the fabric water vapor permeability method commonly employed in the textile industry. The average water vapor permeability of the meso‐hybridized SF/WPU film is 14.95 µg h^−1^, significantly higher than that of polytetrafluoroethylene (PTFE) membrane (≈3.03 µg h^−1^), polyethylene (PE) (≈1.89 µg h^−1^), WPU (≈11.86 µg h^−1^), and TPU (≈12.57 µg h^−1^). This indicates that the meso‐hybridized SF/WPU film is more suitable for prolonged contact with human skin. The results demonstrate that the water vapor permeability of the SF/WPU composite film is superior to that of other polymer films.

Biocompatibility is a critical factor in ensuring the long‐term wearability of wearable devices.^[^ ^26^ ^]^ The SF/WPU films we prepared are highly transparent and ultra‐thin, allowing excellent adhesion to the skin (Figure S3C, Supporting Information), and providing a controllable, flexible material foundation to enhance the comfort of Mo‐Au FMs. Furthermore, cytotoxicity tests were conducted to compare the dry Mo‐Au FM electrode with Ag/AgCl gel electrodes. As shown in Figure S3F (Supporting Information), the experimental results indicated no significant differences in cell viability between the three film‐soaking solutions and the blank control after 5 days of cell culture, as reflected in the absorbance values representing cell content.

Regarding skin‐friendliness, both types of electrodes were worn in direct contact with human skin for one week (Figure S4A, Supporting Information). Significant skin inflammation and irritations were observed with the commercial wet electrodes on the wrists, whereas no notable issues occurred with the continuous application of the Mo‐Au FM electrode. These results indicate that the Mo‐Au FM electrode displays outstanding biocompatibility and skin‐friendliness, making it ideal for long‐term health monitoring without causing harmful or irritating effects on the wearer's skin.

To characterize the sensitivity of the Mo‐Au FM, we present the relative change in resistance (*ΔR*/*R^0^*) values in Figure 3D. The gauge factor (GF) values of the flexible sensors are the slopes of the sensors' (*ΔR*/*R^0^*) relative to the strain curve.^[^ ^27^ ^]^ The detection range of the Mo‐Au FM is from 5% to 25% strain. With the 0% to 5% strain range, the GF of the Mo‐Au FM is 8.4. In the 5% to 15% strain range, the GF is 3.33. Within the 15% to 25% strain range, the GF is 1.8. The Mo‐Au FM electrodes exhibit low skin electrical impedance.^[^ ^13b^ ^]^

Two circular Mo‐Au FM electrodes, each 3 cm in diameter, were placed 10 cm apart on a volunteer's forearm. The impedance of the Mo‐Au FM electrodes slightly decreased after treatments with grease, artificial perspiration, and ethanol, and after being stretched thousands of times (Figure 3C). This reduction in impedance is attributed to the high conductivity and skin‐gripping properties of the Mo‐Au FM electrodes, which outperform commercial Ag/AgCl gel electrodes. The Mo‐Au FM electrodes exhibit lower impedances compared to the Ag/AgCl gel electrodes (Figure 3F‐i‐ii). For instance, a 26‐year‐old male showed impedance values of 37 kΩ cm^2^ for the Mo‐Au FM and 61 kΩ cm^2^ for Ag/AgCl gel electrodes at 10 Hz. Similarly, a 28‐year‐old female had impedance values of 25 kΩ cm^2^ and 36 kΩ cm^2^ at 10 Hz for the Mo‐Au FM and Ag/AgCl gel electrodes, respectively.

Using 1/EIS to represent conductance in EIS measurements reflects the system's ability to conduct electricity under alternating current conditions. In Figure 3E, compared to electrodes utilizing metal‐plated nanolayers,^[^ ^28^ ^]^ CNT‐COOH,^[^ ^12a^ ^]^ carbon black/rGO,^[^ ^29^ ^]^ Ppy,^[^ ^30^ ^]^ PEDOT: PSS,^[^ ^13c^ ^]^ commercial no‐gel electrodes,^[^ ^2b^ ^]^ and gel electrodes, our Mo‐Au FM electrodes demonstrate remarkably higher 1/EIS or lower skin‐contact impedance. If 1/EIS is used to characterize electrode performance, Figure 3E shows that the operation duration of the Mo‐Au FM electrodes is 120 times longer than other high‐performance dry electrodes.^[^ ^13c^ ^]^ Furthermore, the impedance of the Mo‐Au FM electrodes on the skin remains stable during various activities, including lying, standing, walking, running, and during sweat secretion (Figure 3F‐iii‐vi). The unusual performance can be attributed to the intrinsic rigidity of Mo filaments. This long‐term stability in impedance, combined with their skin‐gripping properties, makes the Mo‐Au FM electrodes suitable for prolonged healthcare monitoring applications.

### 2.4 ECG Motional Sensing Performance

**Figure 4:**
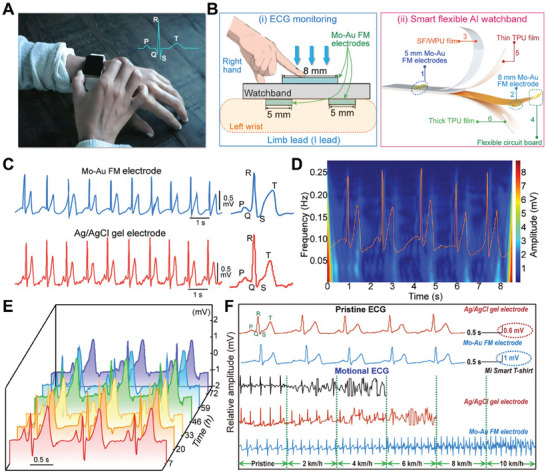
The measurement of ECG at various states by Mo‐Au FM electrodes, compared with commercial Ag/AgCl gel electrodes. A) Depiction of a subject wearing an ECG monitoring watchband on the left wrist. The illustration includes a real‐time electrocardiogram waveform showcasing the R, P, Q, and S waves, demonstrating the device's capability for continuous cardiac monitoring. B) (i) Cardiac electrical signals are monitored by touching the right hand to the surface of the watchband, where an 8 mm diameter Mo‐Au FM electrode is located. (ii) Schema of the smart flexible watchband: 1: Flexible dry Mo‐Au FM electrodes with a diameter of 5 mm. 2: Flexible dry Mo‐Au FM electrodes with a diameter of 8 mm. 3: Water vapor permeability and skin‐friendly meso hybridized SF/WPU film. 4: Flexible circuit board. 5:Thin TPU film. 6: Thick TPU film. C) The comparison of pristine ECG signals of Mo‐Au FM dry electrodes with Ag/AgCl gel electrodes. D) The spectrogram was recorded by the dry Mo‐Au FM electrode. E) The ECG for long‐term (72 h) using Mo‐Au FM dry electrodes and their RMS noise. F) The comparison of motional ECG signals for various types of electrodes (the commercial Ag/AgCl gel electrode, Mi Smart T‐shirt, and Mo‐Au FM electrode). They were acquired under the pristine and the motional states, at different walking/running speeds (from 4 to 10 km h^−1^).

The Mo‐Au FM electrodes are designed to function as wearable dry electrodes with high sensitivity, SNR, long and sustainable operation capability, and low motion noise when sensing epidermal biopotentials. This paper illustrates a sophisticated wearable technology that incorporates ECG monitoring through a smart AI watchband.^[^ ^13c^ ^]^ **Figure** **4**A depicts an individual adjusting the watchband, equipped with Mo‐Au FM electrodes that are strategically positioned to optimize heart signal capture. Cardiac electrical signals are monitored by touching the right hand to the surface of the watchband, where an 8 mm diameter Mo‐Au FM electrode is located.

Figure 4B provides a detailed schematic of the “Smart Flexible AI Watchband”, identifying key components such as Mo‐Au FM electrodes of varying sizes (5/8 mm), a flexible circuit board, SF/WPU film, and both thin and thick TPU films. These elements work together to enhance the device's flexibility and functionality, showcasing the layering and assembly of the watchband's structure. The biocompatibility, conformability, and skin‐gripping properties of the electrodes minimize the risk of skin irritation, a common issue with many wearable devices. The absence of skin irritation, even after 72 h of continuous wear, further supports their suitability for long‐term health monitoring applications (Figure S5B, Supporting Information).

The high‐quality ECG signals produced by the Mo‐Au FM electrodes exhibit clear PQRST waveforms with a peak‐to‐peak amplitude (QRS complex) of 1.0 mV (Figure 4C). The ability to obtain ECG waveforms with peak‐to‐peak amplitudes comparable to those recorded by standard Ag/AgCl gel electrodes underscores the electrodes' potential for clinical use. Additionally, a spectrogram of the ECG pulse,^[^ ^13c^ ^]^ obtained through Fourier Transformation, is particularly beneficial for clinical diagnostics. Accurate signal interpretation is critical for identifying arrhythmias or heart defects (Figure 4D).^[^ ^13^, ^28^ ^]^

The Mo‐Au FM electrodes are suitable for long‐term, as evidenced by the high‐quality ECG signals obtained after 72 h (Figure 4E). The noise is evaluated using root‐mean‐squared (RMS) analysis, which measures signal fluctuations over time.^[^ ^13c^ ^]^ The RMS noise recorded with the Mo‐Au FM electrodes is ≈18 µV, lower than the 19 µV measured with Ag/AgCl gel electrodes (Figure 4F; Figure S4B, Supporting Information). Moreover, after one week of use, the noise level only increased to 28 µV for the Mo‐Au FM electrodes, compared to 33 µV for the Ag/AgCl gel electrodes. Thus, the Mo‐Au FM electrodes surpass the Ag/AgCl gel electrodes for long‐term monitoring.

When compared to the *Mi* Smart T‐shirt or Ag/AgCl gel electrodes, the Mo‐Au FM electrodes demonstrate superior signal quality during motion. This comparison highlights the progress in wearable electrode technology, with Mo‐Au FM electrodes setting a new standard for performance and reliability in long‐term biopotential monitoring. The long‐term stability, minimal noise increases over time, and superior signal quality of the Mo‐Au FM electrodes, combined with their excellent skin gripping properties, make them a highly alternative to traditional gel electrodes.

### 2.5 EMG Sensing and Gesture Signaling

The Mo‐Au FM electrodes have been tested on various muscle groups.^[^ ^28^, ^31^ ^]^ For example, the Mo‐Au FM electrodes were placed on the anterior tibial muscles of a 30‐year‐old volunteer's leg, as depicted in Figure S4E (Supporting Information). During dorsiflexion, the anterior tibial muscles contract and generate EMG signals (Figure S4F, Supporting Information).

**Figure 5:**
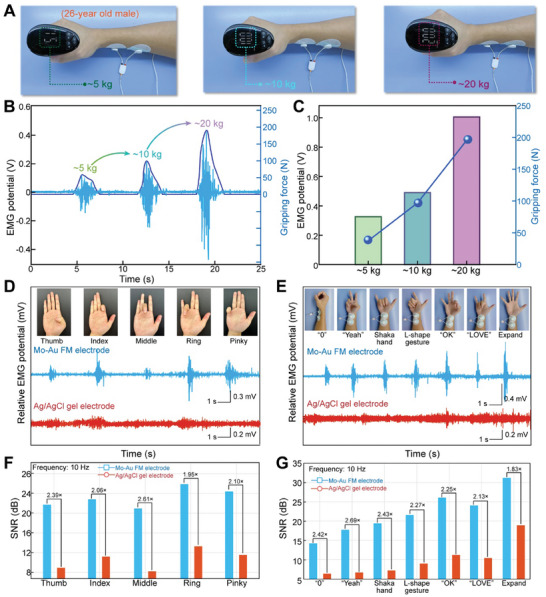
EMG sensing applications based on smart AI watchband with Mo‐Au FM electrodes. A) The measurement of the EMG signals on a forearm by gripping force of ≈5 kg, 10 kg, and 20 kg, respectively. B‐C) EMG signals while gripping the dynamometer. The gripping force can be measured quantitatively. The sensitivity of Mo‐Au FM electrodes is 44.43 mV kg^−1^. The SNR is 43.22 dB. D) The comparison of EMG signal intensities between the gel electrode and Mo‐Au FM electrodes, made by the flexion/extension of different fingers (Thumb, Index, Middle, Ring, and Pinky). E) The comparison of EMG signal intensities between the gel electrodes and Mo‐Au FM electrodes made by the different gestures (“0”, “Yeah”, “Shakehand”, “L‐shape gesture”, “OK”, “LOVE”, and “Expand”). F) The comparison of SNR value between the gel electrodes (orange‐red bars) and Mo‐Au FM electrodes (light blue bars), made by the flexion/extension of different fingers. G) The comparison of SNR value between the gel electrodes (orange‐red bars) and Mo‐Au FM electrodes (light blue bars) made by the different gestures (“0”, “Yeah”, “Shakehand”, “L‐shape gesture”, “OK”, “LOVE”, and “Expand”).

In another scenario, a 26‐year‐old volunteer placed two Mo‐Au FM electrodes on the wrist flexor muscles (inner side of the forearm) (refer to Figure S4B‐D (Supporting Information) for details).^[^ ^12b^ ^]^ We quantitatively measured the amplitude of bipotential relative to the grabbing force. When gripping a dynamometer at various forces (≈5, 10, and 20 kg), the wrist flexor muscles generated corresponding EMG signals (**Figure** **5**A). A linear correlation was observed between the strength of the grip and the amplitude of the EMG signals, as evidenced in Figure 5B,C. The watchband translates EMG signals from arm movements into real‐time control signals, precisely mapped onto the robot, enabling intuitive performance of complex tasks such as deep‐sea exploration or remote operations. The display screen shows EMG signal strengths ranging from 5 to 20 kg, revealing the device's fine‐grained ability to discern variations in grip strength. This mode of HMI enhances operational precision, underscoring the significant role of flexible electronics in the future of collaborative HMI. Under current watchband conditions, the SNR of the Mo‐Au FM electrodes is 43.22 dB at a frequency of 10 Hz, significantly higher than the 15.83 dB of traditional Ag/AgCl gel electrodes (Figure S4G, Supporting Information).

In HMI, hand gestures are a popular method for controlling machines. The EMG signals produced during finger movements, shown in Figure 5D,E and Figure S4C‐D (Supporting Information), highlight the electrodes' sensitivity to subtle muscular activities. The sensitivity of Mo‐Au FM electrodes is 44.43 mV kg^−1^, which is crucial for applications requiring fine‐grained muscle monitoring, such as rehabilitation therapy or nuanced control of prosthetics. Figure 5F,G provide the detailed SNR of Mo‐Au FM electrode and Ag/AgCl gel electrode for subtle muscle activity at 10 Hz. The average SNR of the Mo‐Au FM electrodes for finger‐bending is approximately 2.5 times higher than that of the gel electrodes.

To ensure the reliability and durability of the Mo‐Au FM electrodes, their performance under various conditions was assessed and compared with gel electrodes. Evaluations include immersion in artificial perspiration, ethanol, grease, exposure to salty spray, and repeated stretching (thousands of times) (Figure S5, Supporting Information). These assessments simulate environments the electrodes may encounter during actual use. Under these conditions, the Mo‐Au FM electrodes exhibited excellent corrosion resistance, low contact impedance, and strong skin‐gripping properties, with more stable performance than the gel electrodes. Additionally, the Mo‐Au FM electrodes demonstrated a longer lifespan and more consistent performance in practical applications. This research is crucial for developing a reliable smart watchband system, which typically needs to operate in various complex environmental conditions while maintaining stable performance over extended periods.

### 2.6 Application of Deep Learning to Gesture Recognition

**Figure 6:**
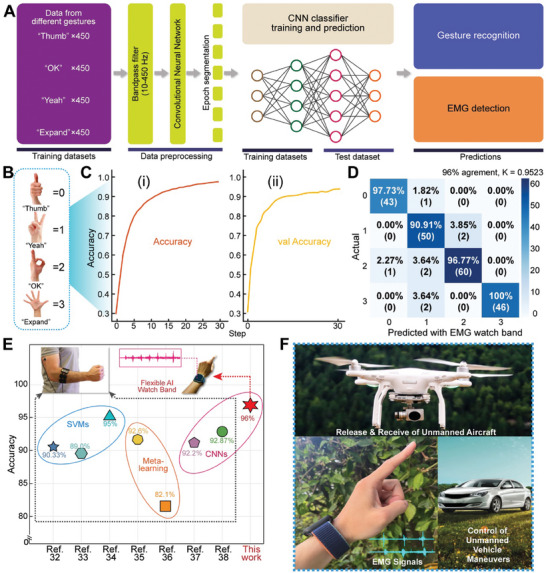
Deep learning on gesture recognition based on the smartwatch band of multi‐channels of Mo‐Au FM electrodes and the potential applications. A) The flow chart illustration for an overview of the data processing and deep learning implementation for gesture classification. The device's data from 20 healthy subjects. B) The collected types of gestures and corresponding identifiers. C) The outcomes of model training. (i) Through training on the acquired data, our model achieved an accuracy rate of 98% on the training set; (ii) Our model demonstrated reaching an accuracy rate of 96% (testing set). D) The performance of the CNN algorithm shows a high agreement (96% and Cohen's kappa value = 0.9523). E) The accuracy (test set) of geatures in this work in comparison with those (test set) of reported cases achieved using different types of algorithms for hand gestures. These algorithms include SVMs, CNN, and Meta‐learning pre‐trained on EMG signals. F) The potential applications of the smart flexible watch band, integrated with drone formation control, will allow the operation to intuitively command drone flights and release unmanned aircraft from a carrier. It can also be applied to implement maneuvers such as unlocking, autopiloting, auto‐parking, etc., by gesture controls.

Compared to ECG, sensing EMG signals is challenging due to their weak strength and susceptibility to noise interference.^[^ ^2^, ^32^ ^]^ The integration of Mo‐Au FM electrodes into a smart flexible watchband for gesture recognition through EMG signal analysis represents a significant advancement in HMI. The workflow depicted in **Figure** **6**A outlines the process from collecting EMG data from various hand gestures to classifying them using a CNN. The application of bandpass filters, epoch segmentation, and CNN classification forms a robust method for processing and interpreting EMG signals, with SNR playing a crucial role. Gesture data are fed into the CNN, and high‐quality data are crucial for accurate gesture recognition.

To further evaluate the adaptability of the smart flexible watchband system under different exercise conditions and states of perspiration, we conducted comparative tests on the contact impedance between dry Mo‐Au FM electrodes and wet electrodes. These tests were carried out under conditions including artificial sweat, salt spray treatment, oil treatment, ethanol treatment, repeated bending, and prolonged wear. The smart flexible watchband, worn by a volunteer (25‐year‐old), was used to measure the EIS. It follows that the EIS of the smart flexible watchband with dry Mo‐Au FM electrodes is lower than that of wet electrodes, leading to higher sensitivity and SNR. This gives rise to high‐quality gesture data, providing a solid foundation for precise ECG/EMG testing in complex environments (Figure S4, Supporting Information).

To establish a comprehensive deep learning workflow, Figure 6A outlines the process from data acquisition and preprocessing to model training, evaluation, and final prediction. This highlights the transformation from raw data to practical model applications and emphasizes the crucial techniques and methodologies employed at each stage.^[^ ^33^ ^]^ Furthermore, Figure 6B provides a detailed depiction of the collected gesture types and their corresponding identifiers. Each gesture undergoes meticulous definition and categorization to ensure consistency and precision during data collection, thereby providing a solid foundation for high‐quality data crucial for subsequent model training and prediction.^[^ ^34^ ^]^ In terms of training on the acquired data, our model achieved an accuracy rate of 98% on the training set (*n* = 1800) (as illustrated in Figure 6C‐i). To validate its robustness in real‐world scenarios, the model also demonstrated remarkable performance on the validation set, achieving an accuracy rate of 96% (*n* = 207) (as depicted in Figure 6C‐ii). These results underscore the efficacy of our model and the long‐term stability, minimal noise increases over time, and superior signal quality, combined with the skin‐gripping properties of our watchband. Figure 6D presents a comprehensive summary of the performance metrics of our CNN algorithm, including overall auto‐recognition consistency and Cohen's kappa coefficient (0.9523), further highlighting the strong performance.^[^ ^35^ ^]^ This reaffirms the immense potential and practical applicability of deep learning in the domain of gesture recognition.

To compare the accuracy of deep learning methods, we evaluated the results obtained from the CNN algorithm based on EMG signal analysis in this work, with traditional algorithms like support vector machines (SVMs) for signal analysis, as well as CNN and Meta algorithms based on image analysis (Figure 6E). SVMs are powerful classifiers that have been successfully applied to hand gesture recognition using surface EMG (sEMG) signals. Previous studies have reported high accuracy, with one achieving 99.37% on the training set and 90.33% on the test set,^[^ ^36^ ^]^ and another study reporting 89.0% accuracy.^[^ ^37^ ^]^

Another HMI approach is gloved‐based. Wen et al. applied a superhydrophobic triboelectric glove by coating it with CNTs and thermoplastic elastomer and applying it in AR/VR environments. This glove‐based HMI demonstrated 95% accuracy in recognizing complex gestures involving all 10 fingers, allowing the execution of various hand tasks, such as flower arrangement in an AR application.^[^ ^38^ ^]^

Meta‐learning is another essential example of adaptive learning, as it can be rapidly fine‐tuned for new tasks. Tan et al. reported a hand gesture recognition wristband using hybrid generators (TENG and PENG) to distinguish between strong force and slight contact.^[^ ^39^ ^]^ This system achieved a 92.6% accuracy for recognizing 26 different gestures.^[^ ^39^ ^]^ A virtual keyboard based on silver‐gold core‐shell nanowires, integrated with a meta‐learning system, was also developed to enable object recognition through surface rubbing, with an accuracy of 82.1%.^[^ ^40^ ^]^ A microcrack‐based bending sensor on a PDMS was developed for joint monitoring, tracking finger bending with 92.2% accuracy using random forest.^[^ ^41^ ^]^ Moin et al. created a system that used sEMG signals captured by conductive Ag ink on a PET substrate to classify 21 hand gestures with 92.87% accuracy, independent of finger count.^[^ ^42^ ^]^

Overall, compared to traditional machine learning algorithms, deep learning models, especially CNNs, are better suited for capturing complex features and patterns in data and exhibit stronger generalization abilities on large‐scale datasets. In contrast, traditional machine learning algorithms like SVMs often encounter performance bottlenecks when dealing with high‐dimensional and large‐scale datasets, as they require manual feature selection and extraction and lack scalability in terms of dataset size and feature space. Consequently, deep learning approaches are more suitable for handling complex data and tasks, leading to higher accuracy and performance. In this study, we employed CNNs to recognize gestures based on EMG signals. A training dataset was constructed using 20 volunteers, with each participant repeating 25 instances of the “Thumb”, “OK”, “Yeah”, and “Expand” gestures. Bandpass filters, with frequencies ranging from 10 to 450 Hz, were used to extract the relevant EMG signal features. After thorough model training and parameter tuning with CNNs, we achieved excellent gesture recognition results, underscoring the effectiveness of CNNs in EMG signal analysis for gesture recognition applications.

Integrating smart gesture‐recognition watch bands, unmanned vehicles, and aircraft carrier control systems represents a pivotal convergence of cutting‐edge technologies, poised to redefine the landscape of HMI. At the forefront of this revolution lies unmanned vehicles, where gesture control emerges as a transformative interface. Unlike conventional control methods that rely on cumbersome input devices or complex interfaces, gesture control offers operators an intuitive and seamless engagement. By simply gesturing, operators can effortlessly navigate intricate environments, adjust vehicle parameters in real‐time, and fine‐tune navigation systems with unprecedented ease. This not only enhances operational convenience but also elevates vehicle safety by enabling swift responses to emergencies or changing conditions, mitigating risks, and ensuring mission success.^[^ ^41^ ^]^ Furthermore, the integration of gesture control technology on aircraft carriers represents a paradigm shift in flight deck operations. Traditionally, carrier operations have relied heavily on physical interfaces and manual inputs, which are labor‐intensive and prone to human error. Gesture control simplifies complex tasks, such as takeoff and landing, streamlining operations.^[^ ^40^ ^]^ By replacing physical buttons and consoles with intuitive gestures, operators can execute tasks more efficiently, reduce cognitive load, and improve situational awareness, ultimately improving operational effectiveness. Gesture control technology also facilitates space‐saving and equipment simplification on aircraft carriers, optimizing resource utilization and enhancing flexibility (Figure 6F). Despite its clear advantages, the widespread adoption of gesture control technology faces challenges, including precision requirements, real‐time responsiveness, and user learning curves. However, advancements in sensor technology, machine learning algorithms, and HMI design are steadily addressing these hurdles. Standardizing gesture control protocols and interfaces is also underway, enhancing interoperability and usability across platforms and applications. Looking ahead, the future of gesture control technology appears increasingly promising. As sensor technology evolves and becomes more affordable, gesture recognition will become more ubiquitous, permeating various aspects of daily life and industry. From automotive interfaces and consumer electronics to healthcare and industrial machinery, the applications are limitless. By empowering users with natural, intuitive interaction methods, gesture control technology has the potential to revolutionize human‐machine collaboration, unlocking new levels of efficiency, safety, and convenience across diverse domains. Continued development and integration of gesture control technology are pivotal steps toward realizing a more interconnected and intelligent future.

## 3 Conclusion

In conclusion, the Mo‐Au FM electrodes demonstrate exceptional performance for biopotential detection. The integration of advanced materials, innovative manufacturing techniques, and state‐of‐the‐art deep‐learning technologies marks the beginning of a new era in wearable health monitoring. This development holds considerable potential for improving home healthcare, digital health monitoring, and quantitative disease diagnosis, marking a transformative advancement in biomedical engineering.

The smart flexible watchbands exhibit low impedance, high conductivity, and outstanding durability, surpassing commercial Ag/AgCl gel electrodes and other stretchable biopotential electrodes found in the literature. Their stable impedance over extended periods makes them ideal for prolonged healthcare monitoring. The skin‐gripping properties of the Mo‐Au FM electrodes ensure consistent contact with the skin, enhancing the accuracy and reliability of long‐term biopotential recordings. The integration of these electrodes into a smart flexible watchband system has resulted in a comprehensive deep‐learning workflow for personalized health management. This system offers a user‐friendly and convenient solution for the real‐time acquisition and recording of physiological electrical activity.

Advanced algorithms, such as CNN, enable accurate predictions and classifications of gestures. The robustness and efficacy of the developed system are demonstrated through high accuracy rates in training and validation sets, highlighting the potential of deep learning in gesture recognition applications. Combined with the high performance of flexible Mo‐Au FM electrodes, the smart watchbands exhibit superior gesture recognition accuracy, long‐term wearability, and sustainable application.

Overall, the integration of Mo‐Au FM electrodes with a smart flexible watchband system showcases their versatility and practical value in personalized healthcare monitoring. This advancement paves the way for improved diagnostics, data analysis, and gesture recognition capabilities. Future developments in these areas hold great promise for enhancing health management and contributing significantly to the field of wearable biosensing devices.

## 4 Experimental Section

### Preparation of SF/PU Film

The silk fibroin solution was prepared in the previous work.^[^ ^21g^ ^]^ The SF and WPU solutions (both 10 wt.%) were mixed and stirred for 1 h at room temperature. The mixture was then drop‐casting into a petri dish and dried at 60 °C for 2 h before the SF/PU films were peeled off. Finally, the resultant SF/PU films were peeled off.

### Cell Culture

Human immortalized epidermal cells (HACAT) and Human umbilical vein endothelial cells (HUVEC) were obtained from the Emergency and Trauma College of Hainan Medical University (Hainan, China). HACAT and HUVEC cells (from ATCC) are cultured in minimum essential medium DMEM, supplemented with 10 vol % fetal medium bovine serum (FBS), l vol % antibiotics penicillin‐streptomycin in an incubator at 37 °C, 70–80% relative humidity to observe the viability by the light microscope. The cells are seeded on the sterilized samples (3 × 3 × 1 mm) and placed in a 96‐well culture plate at a density of 2 × 10^5^ cells per well. After culturing for l, 3, and 5 days, the cell viability is determined using a CCK‐8 assay. The entire experiment is carried out in a sterile laboratory environment.

### Data Processing

EMG signals were collected from 20 volunteers as they performed four distinct hand gestures: “Thumb,” “OK,” “Yeah,” and “Expand,” with each gesture repeated 25 times, resulting in 450 samples per gesture. The raw EMG signals were preprocessed using a bandpass filter with a frequency range of 10–450 Hz to remove noise and artifacts. After filtering, the data were segmented into fixed‐length epochs to capture temporal features across different time windows, facilitating better feature extraction. Next, Standard Scaler was applied for normalization, which standardizes the data by transforming it to have a mean of 0 and a standard deviation of 1. This normalization process ensured that the model could effectively train without being influenced by variations in signal amplitude.

### Machine Learning

A CNN is implemented to classify gestures based on the processed EMG signals. The CNN architecture was designed to learn spatial‐temporal patterns from the segmented signal epochs. The network included several convolutional layers for feature extraction, followed by max‐pooling layers to reduce dimensionality, and fully connected layers for classification. The model was trained using categorical cross‐entropy loss and the Adam optimizer. Another dataset (n = 207) was used for testing, with a validation split to monitor performance during training and prevent overfitting. Both training accuracy and validation accuracy (val accuracy) were tracked to evaluate the model's learning and generalization capabilities. Cohen's Kappa coefficient was calculated to assess the agreement between the predicted and true classifications.

### All Volunteers Provided Informed Written Consent

The first electrode design was tested without any skin preparation or conductive paste as required for the conventional electrode. The results were compared to those obtained with a commercial one, human trials with volunteers.

## Conflict of Interest

The authors declare no conflict of interest.

## Supporting information



Supporting Information

## Data Availability

The data that support the findings of this study are available from the corresponding author upon reasonable request.
