# Supplementary material for: Meso Hybridized Silk Fibroin Watchband for Wearable Biopotential Sensing and AI Gesture Signaling
Source: Adv Sci (Weinh). 2024 Dec 11;12(5):2410702. doi: 10.1002/advs.202410702 (PMC11792041; doi:10.1002/advs.202410702)
Supplement: Supplementary file 1 — Supporting Information [file ADVS-12-2410702-s001.docx]

*Supporting Information*

Meso Hybridized Silk Fibroin Watchband for Wearable Biopotential Sensing and AI Gesture Signaling

*Xiao Wang,^1^† Changsheng Lu,^1^† Zerong Jiang,^1^ Guangwei Shao,*^2^ Jingzhe Cao,^3^ Xiang Yang Liu,^1^**

X. Wang, C. S. Lu, Z. R. Jiang, X. Y. Liu

State Key Laboratory of Marine Environmental Science (MEL)

College of Ocean and Earth Sciences

Xiamen University

Xiamen, Fujian, 361102, P.R. China

G. W. Shao

College of Textiles

Donghua University

Shanghai, 201620, P.R China

J. Z. Cao

College of Textile and Garment

Shaoxing University

Shaoxing, Zhejiang, 312000, P.R. China

**E-mail:** Correspondence: [shaogw@dhu.edu.cn](mailto:shaogw@dhu.edu.cn) (G.W. Shao); [liuxy@xmu.edu.cn](mailto:liuxy@xmu.edu.cn) (X.Y. Liu)

**This PDF file includes:**

Supplemental Notes (Note S1 to S4)

Supplemental Figures (Fig. S1 to S5)

Reference (ref.1 to ref. 3)

Note S1: Materials

WPU aqueous dispersion (WPU-3758, 29.5wt%) was supplied by Guangzhou Ruiling. The WPU is used to prepare adhesive blend film. Polydimethylsiloxane (PDMS, Sylard184) curing agents were obtained from Dow Corning Company. All the chemicals were used as received without further purification.

Note S2: Materials Characterization

The process to analyze the structure of SF/PU film using Fourier Transform Infrared Spectroscopy (FTIR) with a Nicolet IN10 spectrometer (Thermo Fisher, USA). Fourier self-deconvolution (FSD) of the infrared spectra covering the amide I region (1580-1720 cm−1). The XRD (Bruker D8 ADVANCE) patterns of films were collected with a beam size of 0.5 mm in the range of 5°-90° and a scanning rate of 2° min−1. The microstructure was obtained by Su-70 thermal field emission scanning electron microscope (SEM). The thickness of the polymer films was determined with an Alpha 500 step profiler. The impedance spectra were taken with an Autolab impedance analyzer with the dual-electrode method in the ranges of 1-104 Hz. The two electrodes were placed on the forearm with a separation of 10cm. The conductivities of the polymer films were measured with a four-point probe setup fitted with Keithley 2400 source/meter. In the conductivities shown in the figures, the error bars represent the standard error.

Note S3: Mechanical Characterization

The tensile measurements were conducted using an Instron Model 5948 Materials Testing System. The load cell is a 100 N load cell, and the uniaxial strain was applied at a ramp rate of 1mm/min. The load cell was calibrated before the testing.

Note S4: Biopotential Signals Extraction

The ECG signals were acquired by placing two Au-Mo@SF/PU electrodes on the inner wrists and a reference electrode on the rear hand. The electrodes were connected to a signal-recording setup processed with a bandpass filter. The EMG tests were conducted by mounting two Au-Mo@SF/PU electrodes on the upper arm or forearms and a reference electrode on the rear hand for the signal generated by the bicipital or brachioradialis muscle, respectively. For the EMG signals collected for finger flexion and extensions, two Au-Mo@SF/PU electrodes were placed on the forearms. Through potential differences between the working electrodes on the object area and the reference electrodes, the biopotential signals (ECG and EMG) are captured by the Biosignals PLUX System. The signal processing algorithms are performed on the collected data using Matlab for fundamental signal analysis (Root-Mean-Square/Spectrogram/Fast-Fourier Transform).

Recent research has identified five distinct levels of structures within SF materials:^[1]^ (1) amino acid sequences, (2) secondary structures, (3) β-crystallites, (4) crystal networks (also known as individual nanofibrils), and (5) nanofibril networks. Among these levels, the last three (crystal networks, and nanofibril networks) play a crucial role in determining the overall performance of the material.^[2]^ The formation of hierarchical structures in SF materials is controlled by protein nucleation, and altering this process leads to meso-reconstruction, impacting macroscopic performance.^[3]^


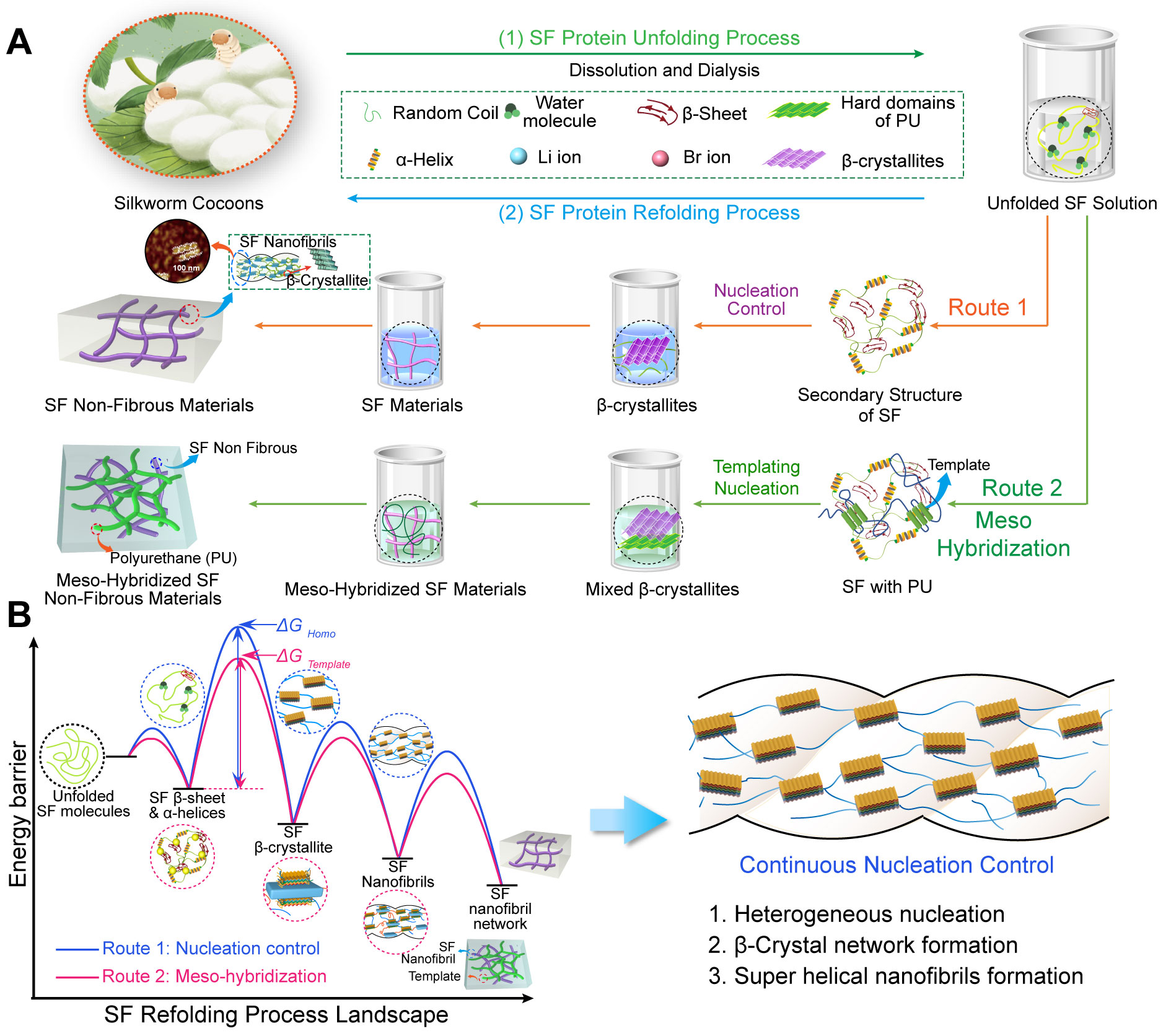


**Figure S1 Meso-reconstruction of SF materials.**

(A) Schematic illustrations of SF protein unfolding process/refolding process. Route 1: Nucleation of β-crystallites with a high energy barrier. Route 2: Nucleation of β-crystallites influenced by templates or seeds.

(B) Schematic illustrations of the crystallization-nucleation mechanism of SF. The crystallization of SF molecules must overcome the so-called nucleation barrier. The high energy barrier can be reduced by introducing nucleation templates or seeds to promote the crystallization of SF molecules into crystallites and the formation of crystallite networks.


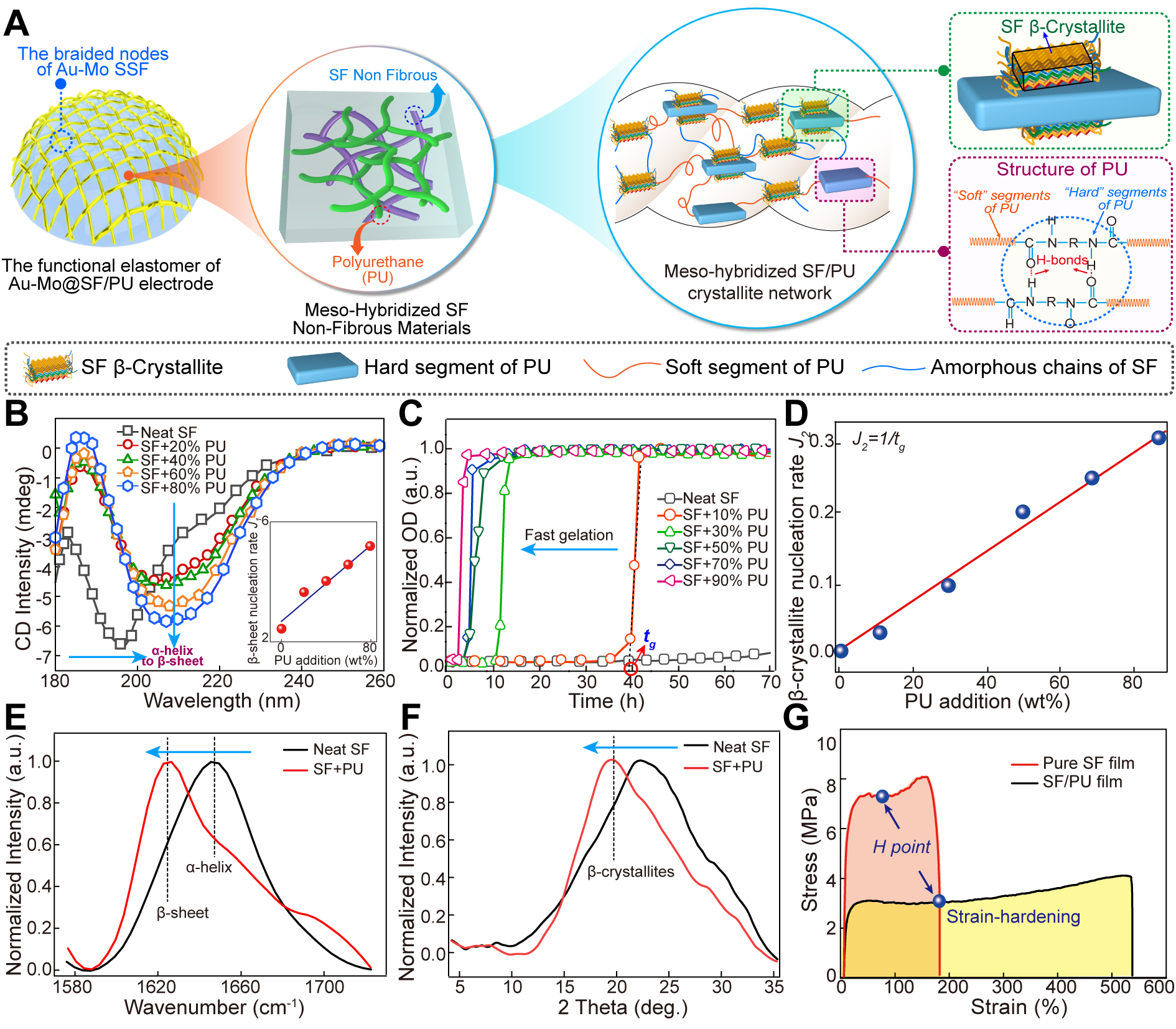


**Figure S2 Meso-reconstruction of SF materials.**

(A) Photographs of the SF/PU flexible film and schematic illustration of the double network structures within meso-hybridized SF/PU materials.

(B) CD spectra of 0.1 mg/ml SF solution containing various quantities of PU. The blue arrow indicates the conformation transition of SF molecules from α-helix/random coil to β-sheet.

(C) Temporal evolution of turbidity in 4wt% SF solutions containing different quantities of PU. The gelation time *t_g_* (defined as the time of abrupt increase) decreased with the addition of PU. The blue arrow indicated the promotion of β-crystallite formation by PU.

(D) The β-crystallite nucleation rate *J_1_* of SF is linearly correlated with the quantities of additive PU.

(E) FTIR spectra of neat SF materials and SF/PU meso-hybridized materials obtained after the same incubation period. The blue arrow indicated the promotion of β-sheet formation by PU templating seeds.

(F) XRD of neat SF materials and SF-PU meso-hybridized materials obtained after the same incubation period.

(G) Typical stress-strain curves of neat SF film and various SF/PU hybrid films incorporated with different quantities of PU.


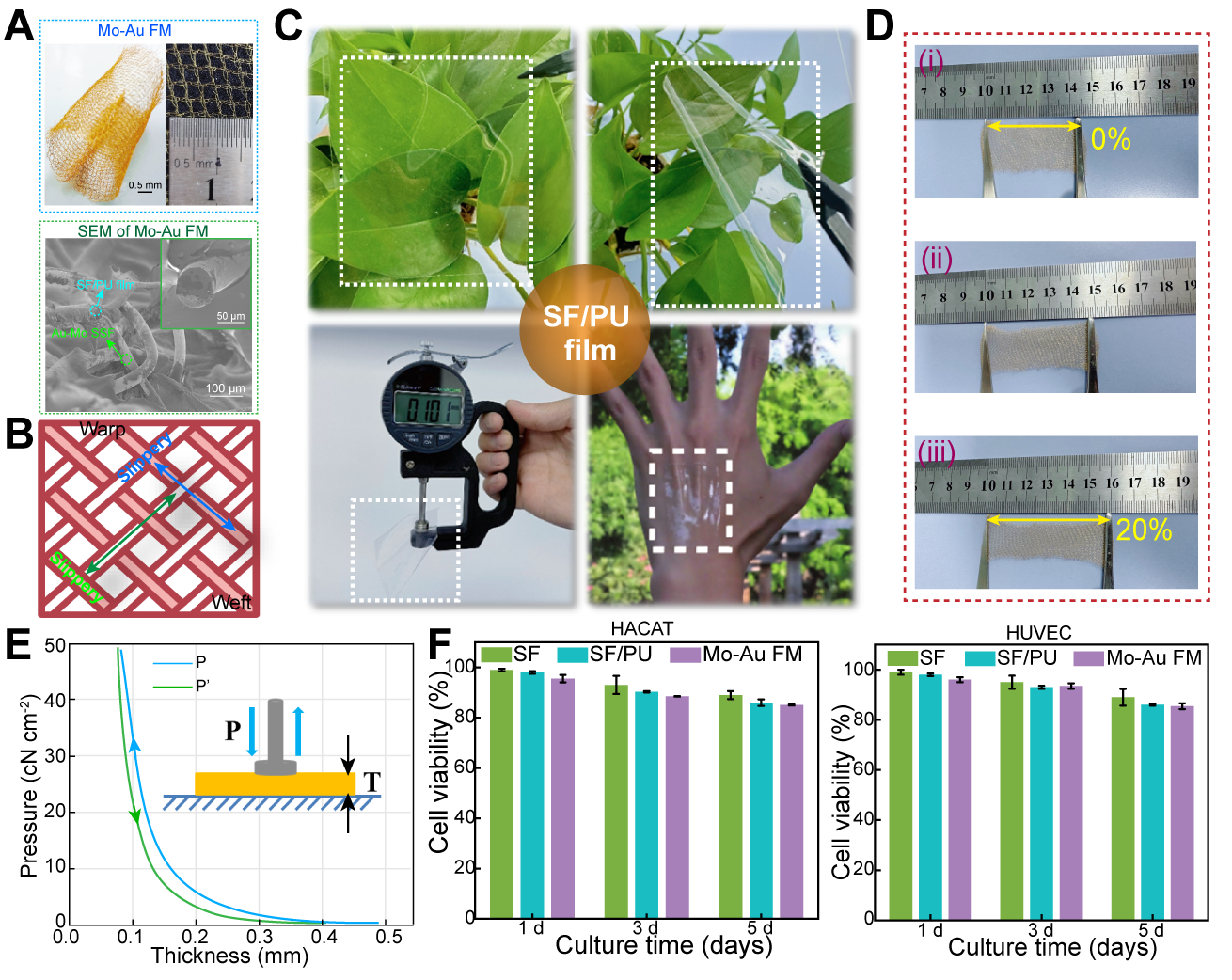


**Figure S3** (A) Image of a hollow tube of Mo-Au FM with dimensions of 3 cm×3 cm in the natural state. And the image of stainless-steel plain cloth. And SEM image of Mo-Au FM and SF/PU film.

(B) Schematic of stainless-steel plain cloth.

(C) Photographs of the SF/PU flexible film and schematic illustration of the double network structures within meso-hybridized SF/PU materials.

(D) Different tensile elongations (0%, 10%, and 20%) of Mo-Au FM.

(E) The resilience of Mo-Au FM. After applying an external force *P*, the thickness of the Mo-Au FM gradually decreases. Subsequently, it relies on its inherent resilience to gradually rebound (*P’*).

(F) The cell viability of the three types of silk-based films by the Human umbilical vein endothelial cells (HACAT) and the Human umbilical vein endothelial cells (HUVEC).


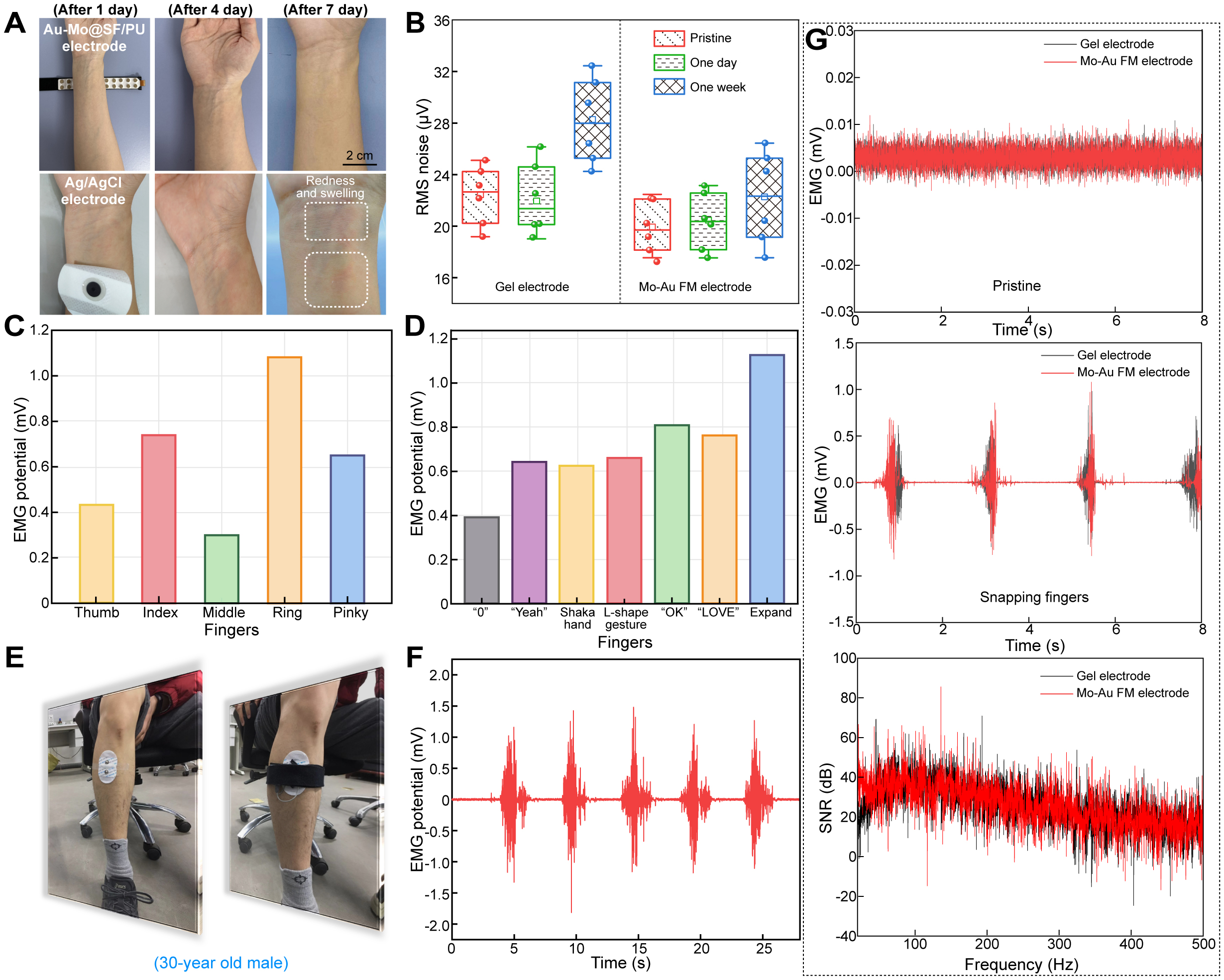


**Figure S4** (A) Comparative analysis of images shows skin inflammation measurements on the human wrist after wearing the Au-Mo@SF/PU electrode and gel electrode after 1, 4, and 7 days. It indicates that Au-Mo@SF/PU electrodes are much more skin-friendly.

(B) The RMS noise was picked by Ag/AgCl gel electrode and Au-Mo@SF/PU dry electrode during ECG recording in pristine, one day, and one week.

(C) EMG signal intensities are produced by the five fingers.

(D) EMG signal intensities are produced by the different gestures.

(E) A 30-year-old male volunteer attaches electrode patches to the tibia position on his lower leg to collect electromyographic (EMG) signals (F) of the calf muscles.

(G) Comparison of EMG signals measured by gel electrode and Mo-Au FM electrode. The top graph shows low-magnitude EMG signals detected during pristine. The middle graph demonstrates the detection of EMG signals generated by snapping fingers. The bottom graph displays the SNR across a frequency range of 0-500 Hz.


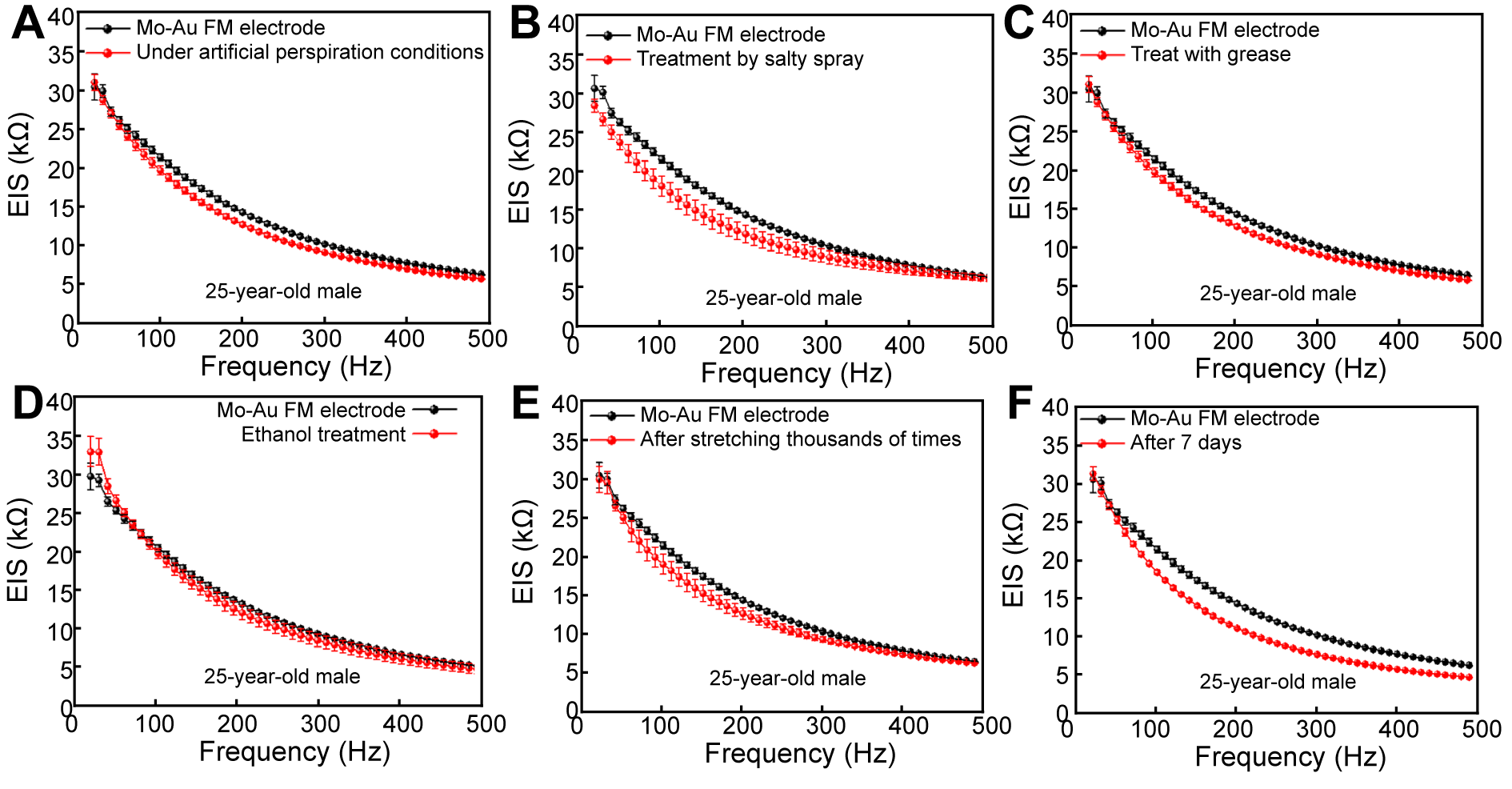


**Figure S5 The electrical performance of Au-Mo@SF/PU electrode.**

(A) EIS comparison between the Au-Mo@SF/PU electrode (red line) and the same electrode under artificial perspiration conditions (black line).

(B) EIS comparison between the Au-Mo@SF/PU electrode (red line) and the same electrode after being treated with salty spray (black line).

(C) EIS comparison between the Au-Mo@SF/PU electrode (red line) and the same electrode after being treated with grease (black line).

(D) EIS comparison between the Au-Mo@SF/PU electrode (red line) and the same electrode after being treated with ethanol (black line).

(E) EIS comparison between the Au-Mo@SF/PU electrode (red line) and the same electrode after being stretched thousands of times (black line).

(F) EIS comparison between the Au-Mo@SF/PU electrode (red line) and the same electrode after 7 days (black line).

**Reference**

[1] W. Qiu, A. Patil, F. Hu, X. Y. Liu, *Small* **2019**, *15*, e1903948.

[2] N. Lin, X. Y. Liu, *Chem. Soc. Rev.* **2015**, *44*, 7881.

[3] W. Qiu, X. Y. Liu, *Adv. Fiber Mater.* **2022**, *4*, 390.
